# Deep learning in breast imaging

**DOI:** 10.1259/bjro.20210060

**Published:** 2022-05-13

**Authors:** Arka Bhowmik, Sarah Eskreis-Winkler

**Affiliations:** ^1^ Department of Radiology, Memorial Sloan Kettering Cancer Center, New York, NY 10065, United States

## Abstract

Millions of breast imaging exams are performed each year in an effort to reduce the morbidity and mortality of breast cancer. Breast imaging exams are performed for cancer screening, diagnostic work-up of suspicious findings, evaluating extent of disease in recently diagnosed breast cancer patients, and determining treatment response. Yet, the interpretation of breast imaging can be subjective, tedious, time-consuming, and prone to human error. Retrospective and small reader studies suggest that deep learning (DL) has great potential to perform medical imaging tasks at or above human-level performance, and may be used to automate aspects of the breast cancer screening process, improve cancer detection rates, decrease unnecessary callbacks and biopsies, optimize patient risk assessment, and open up new possibilities for disease prognostication. Prospective trials are urgently needed to validate these proposed tools, paving the way for real-world clinical use. New regulatory frameworks must also be developed to address the unique ethical, medicolegal, and quality control issues that DL algorithms present. In this article, we review the basics of DL, describe recent DL breast imaging applications including cancer detection and risk prediction, and discuss the challenges and future directions of artificial intelligence-based systems in the field of breast cancer.

## Introduction

Artificial intelligence (AI) has exciting potential to transform the field of medical imaging. Recent advances in computer algorithms, increased availability of computing power, and more widespread access to big data are fueling this revolution. AI algorithms can be taught to extract patterns from large data sets, including data sets containing a vast amount of medical images, and are able to meet, and even exceed, human-level performance in a variety of repetitive well-defined tasks.^1–6^

Breast imaging is particularly well suited for AI algorithm development since the diagnostic question is straightforward and there is widespread availability of data. Most breast imaging exams are binary classification problems (*e.g.* malignant *vs* benign), and almost all studies have an accepted ground truth (*e.g.* histopathology or negative imaging follow-up) that is commonly available for use during algorithmic development. Furthermore, there is widespread availability of standard imaging data due to population-wide screening programs, and the American College of Radiology (ACR) Breast Imaging and Reporting Data System (BI-RADS) system enforces structured reporting and assessments.

To date, retrospective and small reader studies show that AI tools increase diagnostic accuracy,^7–9^ improve breast cancer risk assessment,^10–12^ and predict response to cancer therapy,^13,14^ among other tasks. AI is also being applied to improve the image reconstruction process more generally, so that high quality images may be obtained with lower radiation dose in mammography and digital breast tomosynthesis (DBT), and with shorter scan times in MRI.^15–17^

AI is uniquely poised to help breast imagers at both ends of the interpretive spectrum. On the one hand, AI can be used to automate simple tasks (*e.g.* removing completely normal exams from the radiology worklist, which relieves radiologists to tackle more challenging cases). Computer algorithms do not suffer from fatigue or distraction, and thus are uniquely suited for basic repetitive tasks that humans may find tedious or boring. On the other hand, AI has the potential to extend the frontiers of our practice of medicine. AI can identify complex patterns in imaging data that are not appreciated by the human eye,^10^ adding a wealth of information to enable more sophisticated disease modelling and more individualized treatment planning. However, it is important to note that almost all studies to date have been either retrospective trials or small reader studies, which limits the generalizability of results. Prospective studies are now needed to more fully evaluate the performance of these AI tools, and are prerequisite to responsible clinical translation.

In this article, we will review the basics of AI and deep learning, describe some AI applications in clinical breast imaging, and discuss challenges and future directions.

## Artificial intelligence and DL

Traditional machine learning, a subfield of AI, was used in the 1990s and 2000s to develop computer-aided detection (CAD) software for mammography. In initial studies,^18^ CAD improved diagnostic accuracy, it received FDA approval in 1998, and became widely utilized over the next 18 years.^19^ However, more recently, larger studies demonstrated that CAD generates large numbers of false positives and does not improve diagnostic accuracy, and therefore it has largely fallen out of favor.^20^

**Figure 1.**
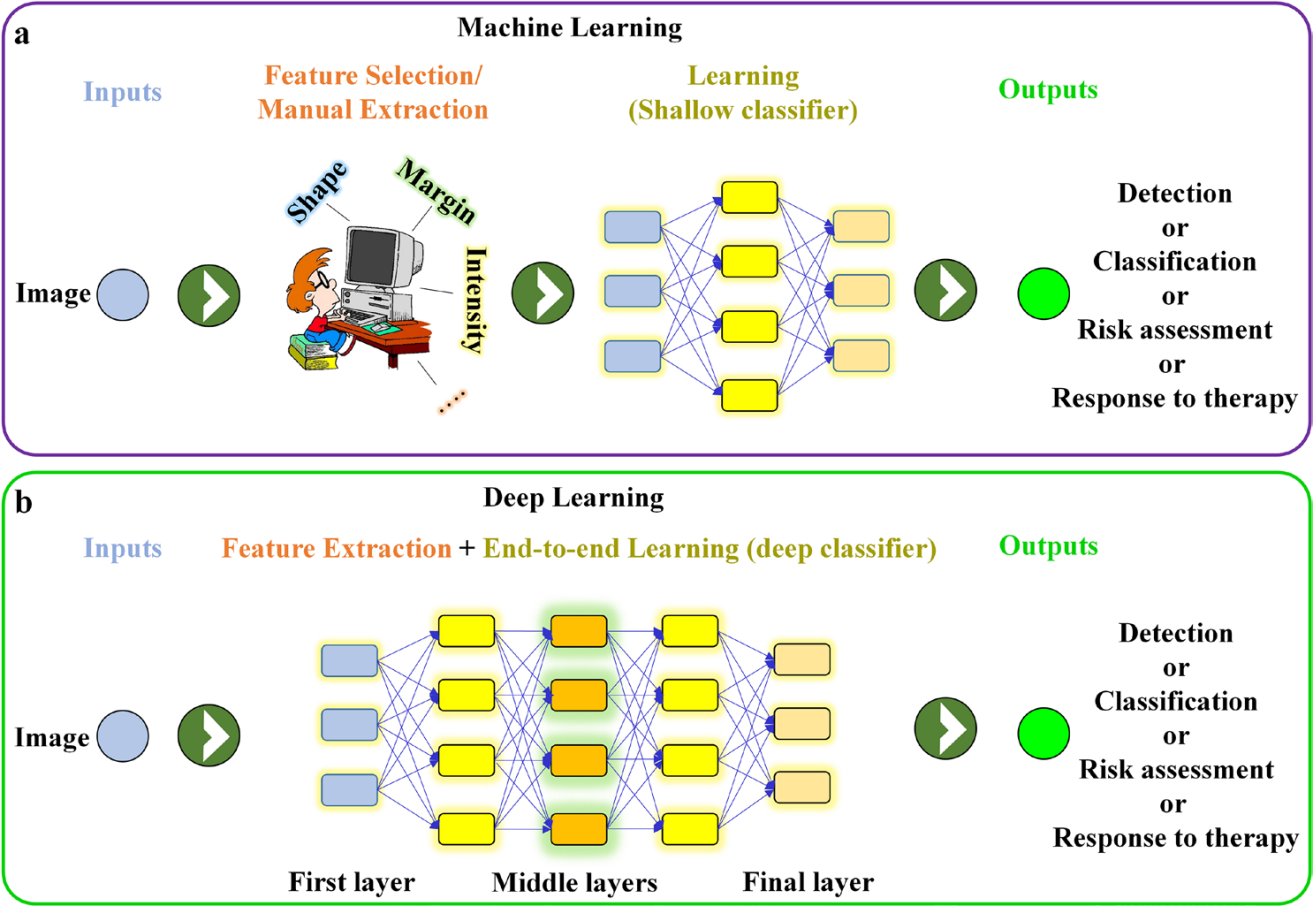
Schematic illustrating (**a**) feature-based (human-engineered) machine learning network (*e.g.* conventional CAD software) and (**b**) end-to-end deep learning network. CAD, computer-aided design.

Deep learning (DL), a new type of representative machine learning, first gained widespread attention in 2012 when AlexNet won the ImageNet Large Scale Visual Recognition Challenge by a large margin.^21^ Since 2016, there has been an explosion of effort in applying DL to diagnostic radiology, and to breast imaging in particular.^22,23^ DL models not only classify input images as positive or negative, but they figure out which imaging features are needed to perform this classification, without expert input.^24^ This is in contrast to traditional machine learning techniques (*e.g.* CAD), which rely on hand-crafted features (*e.g.* shape, margin) to perform the classification (Figure 1a). This major difference explains why new DL algorithms outperform traditional machine learning techniques (if there are sufficient data available).^25^

Most DL algorithms for medical imaging use convolutional neural networks (CNN). CNNs have millions of weights (*i.e.* variables to be optimized) and multiple layers of processing designed to extract hierarchical patterns in data. Most DL models for medical imaging use a supervised learning technique, which means that training is performed using many labeled examples. Data labeling can be done on the exam level (*e.g.* a whole mammogram exam is labeled as benign or malignant), breast level (*e.g.* the left breast is labeled benign and the right breast is labeled malignant), pixel level (*e.g.* the area of malignancy is circled), or somewhere in between. Pixel-level labeling gives the most information and reduces training set size requirements, although it is costly to generate.

During CNN training, a general purpose learning procedure^24^ is used to perform feature selection and classification simultaneously and without expert input (Figure 1b). During CNN training, large numbers of labeled medical images are fed directly to a CNN. The first layer learns small simple features (*e.g.* location and orientation of edges), the next layers learn particular combinations of those simpler features, and the deeper layers learn even more complex arrangements of those earlier patterns. The final layers use these imaging features or representations to classify the image or to detect other patterns of interest (Figure 1b). Once a CNN is fully trained, its performance is tested using a held-out test set not used during the training. Ideally, CNN performance is further validated using a data set from an outside institution (*i.e.* external validation).

DL methods are data-driven and results generally improve as the data set size increases. There is no specific formula to calculate the data set size needed to train a model for a given task, although training data sets must be large enough and diverse enough to encompass the range of phenotypes of the categories that they seek to classify. When it is not possible to curate a data set of sufficient size to train a CNN from scratch (a frequent occurrence in medical imaging), CNN weights may be initialized with weights learned for some other task (*e.g.* classifying cats versus dogs). This transfer learning technique substantially reduces data set size requirements for CNN training.^24,26^

Since DL networks learn complex representations of images not appreciated by the human eye, they have the potential to identify new unseen patterns in data, transcending our current knowledge of disease diagnosis and treatment, although this aspect of DL is still at a pilot stage and warrants further exploration.

## Mammography and digital breast tomosynthesis

### Cancer detection and classification studies

Each year, more than 300,000 cases of breast cancer are diagnosed in the United States alone. While screening mammography decreases breast cancer mortality by 20–35%, it is not a perfect tool.^27^ The diagnostic accuracy of mammography varies widely, even among breast imaging experts, with sensitivity and specificity ranging from 67 to 99% and 71 to 97%, respectively.^28^ DL has the potential to improve these metrics, both increasing cancer detection rates and decreasing unnecessary callbacks. Several retrospective and reader studies have already shown AI model performance at or beyond the level of expert radiologists^9,22,29–31^ (*see* Supplementary Material 1). Reader studies have used a combination of fellowship-trained breast imagers, general radiologists, and sometimes even trainees, which is an important consideration when making claims about the superiority of AI tools compared to human readers. Of note, while initial DL studies were based on 2D mammography, more recent work has focused on DBT,^7,29,32,33^ which is a more complex technical task but which has potential to improve AI performance even further. DBT increases the radiologist’s interpretation time by approximately 50% compared to 2D mammography,^34^ and so AI tools for DBT are being developed not just to find more cancers, but with an eye towards clinical efficiency.

**Figure 2.**
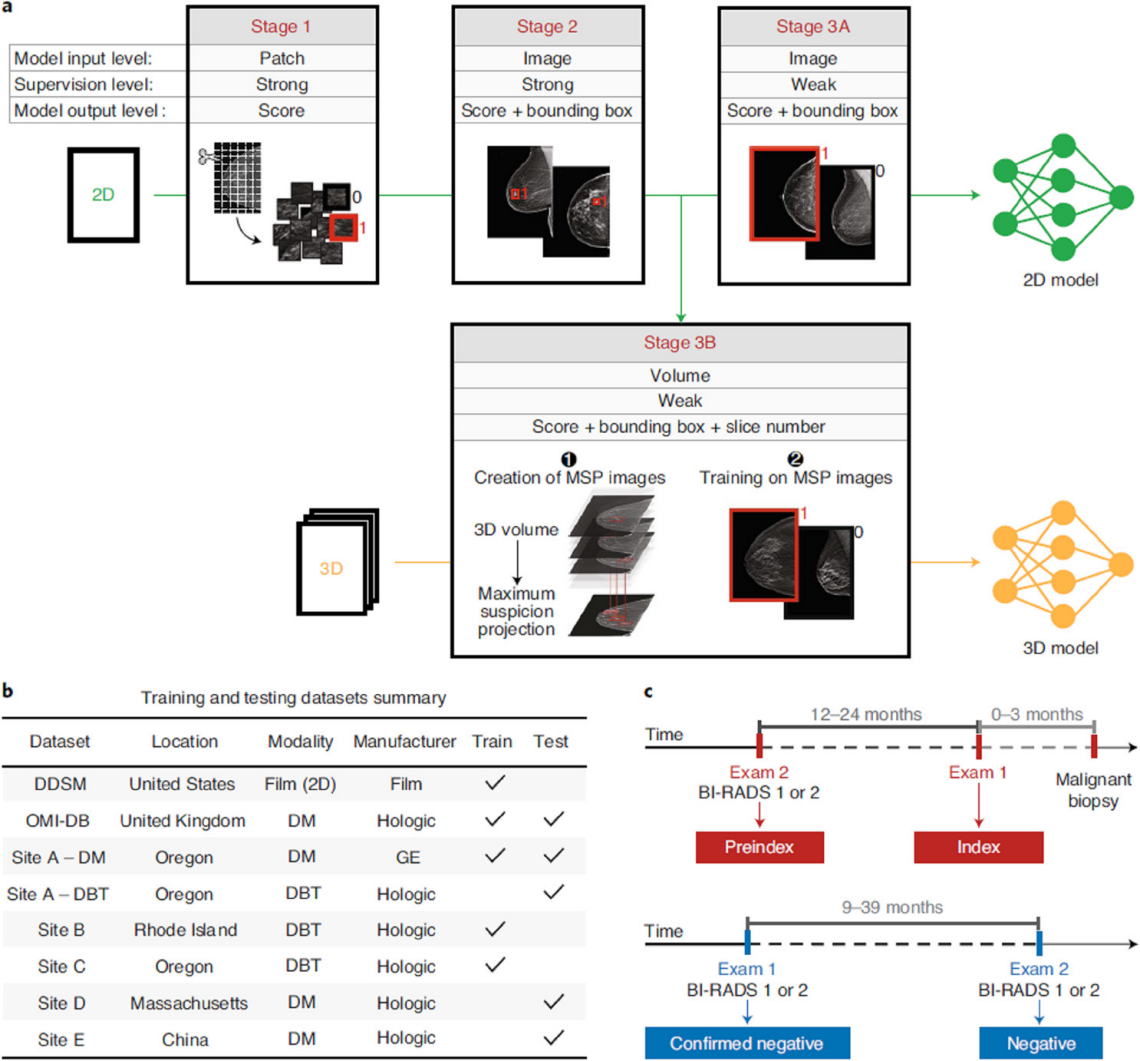
An illustration of multistage deep learning model training and data summary.^29^ (**a**) Illustration of model training in stages. Stage 1 illustrates patch-level classification. Stage 2 demonstrates an intermediate step where a detection-based model was trained on full mammography images to identify bounding boxes and malignancy likelihood scores. Stage 3A re-trains the detection-based model using multi-instance learning approach wherein maximum score over all bounding boxes in each full mammography image was computed for classification of cancer or no cancer. In Stage 3B, an analogous detection-based model was trained for DBT using maximum suspicion projection images. (**b**) Summary of multi-institution training and testing data sets. (**c**) Illustration of exam definitions used in the study.^29^ Reprinted by permission from Springer Nature: Nature Medicine,^29^ copyright 2021.

In one of the most important AI mammography/DBT studies to date, Lotter et al presented a DL model for cancer detection that yielded state-of-the-art performance for mammographic classification, showing an area under the curve (AUC) of 0.945 in their retrospective study.^29^ The DL model outperformed five expert breast imagers in a reader study, worked for both 2D digital mammography and 3D DBT, and was externally validated using imaging data from several national and one international site, demonstrating good generalizability (Figure 2). Another key retrospective study, from Salim et al, independently evaluated performance of three commercial AI systems for mammography screening using a single standardized data set.^35^ One of the three AI algorithms outperformed human readers, and combining that algorithm with a human reader outperformed two human readers.

To pave the way for clinical translation, prospective clinical trials are needed. Several such trials are currently recruiting, most of which are evaluating performance of the Transpara (ScreenPoint Medical) and INSIGHT MMG (Lunit) commercial AI software, in different clinical and geographic settings. For example, the AITIC trial (NCT04949776) will evaluate if Transpara can reduce the workload of a breast screening program by 50% with non-inferior cancer detection and recall rate, whereas the ScreenTrustCAD trial (NCT04778670) will compare the Lunit software to single and double readings by radiologists.

#### Calcifications

Calcifications, a common finding on mammogram, are conventionally classified as suspicious, probably benign, or benign using the ACR BI-RADS lexicon.^36^ However, more than half of calcifications classified as suspicious yield benign pathology,^37^ and so there is much interest in developing DL tools to improve the classification process and avoid unnecessary biopsies. Some groups have demonstrated increases in diagnostic accuracy with DL algorithms, although data set sizes are small and larger validation studies are needed.^38–40^

#### AI for mammography workflow optimization

In the coming years, DL seems poised to transcend its role as a mammography decision-support tool and instead to serve as an independent reader of “ultra-normal” mammograms. Over 20 million^22^ screening mammograms are performed in the United States each year, and over 99% of them are completely normal. If an independent AI reader signed-off on even a fraction of these studies without radiologist input, there could be significant cost savings and impacts on workflow. Several studies^41–43^ have investigated this, with results suggesting that AI could remove up to 20% of the lowest-likelihood-of-cancer screening mammograms from the worklist without missing cancers. Larger trials are warranted to validate these promising findings. Ethical, medicolegal, and regulatory aspects of standalone AI warrant further consideration prior to clinical translation. For example, it will be important to develop AI-specific quality controls, including a schedule for continuous algorithmic assessment and fine-tuning to ensure that AI performance does not drift over time.

#### DL for breast cancer risk assessment

**Figure 3.**
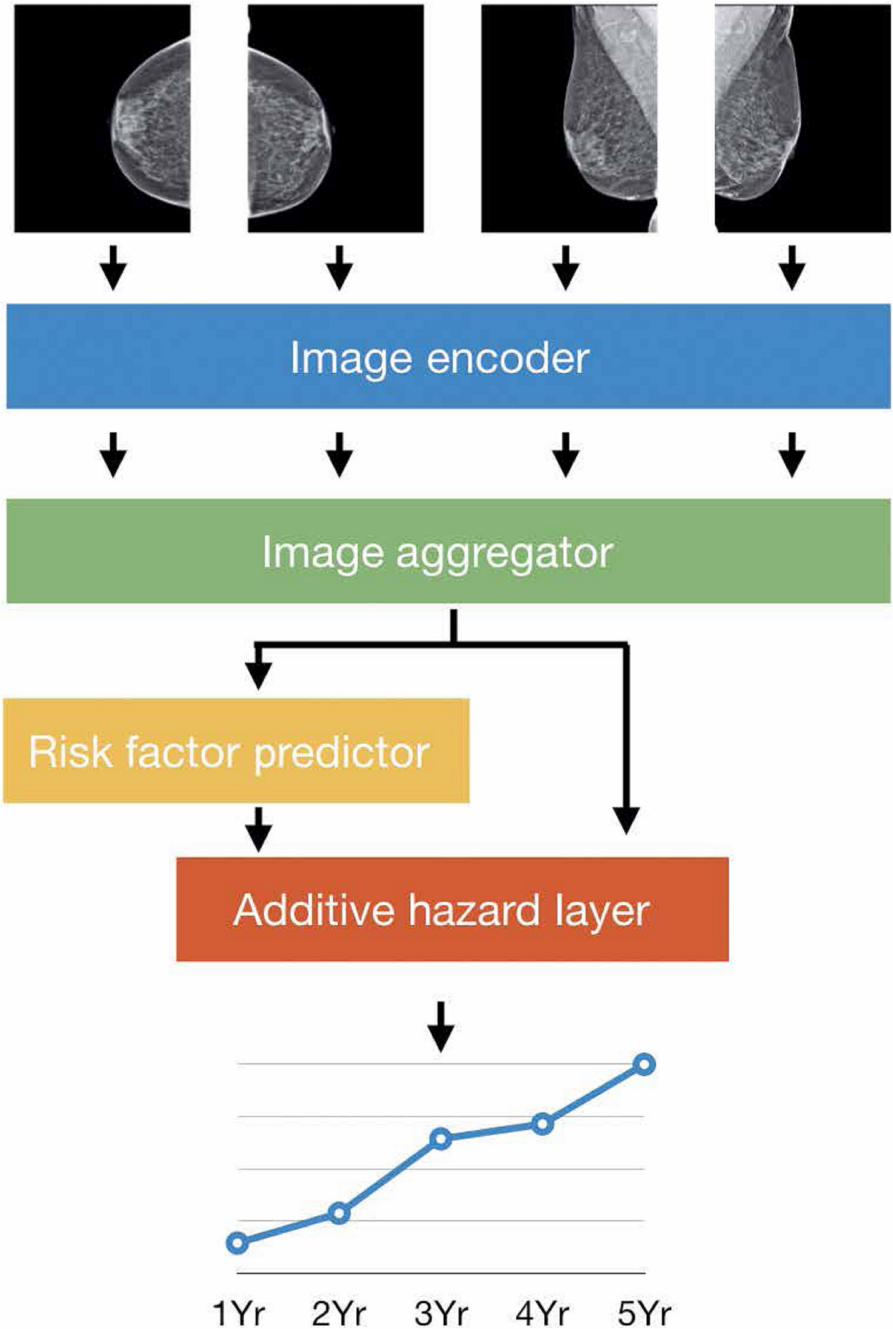
A schematic illustration of breast cancer risk-prediction DL model.^10^ The model input is four standard views of an individual mammogram. The image encoder and image aggregator together provide a combined single vector for all mammogram views. Standard clinical variables (*e.g.* age, family history) are incorporated into the DL model, and if any of these clinical variables are unavailable, a risk factor predictor module is used to fill in the missing pieces. Finally, the additive hazard layer combines the imaging and clinical data to predict breast cancer risk for five consecutive years. Reprinted by permission from The American Association for the Advancement of Science: Science Translational Medicine,^10^ copyright 2021. DL, deep learning.

In one of the most significant AI-for-medical-imaging developments to date, DL has been used in an effort to optimize breast cancer screening practices. At present, conventional risk assessment models such as the Tyrer–Cuzick model are used to determine whether a woman is at high risk of breast cancer, a status which warrants supplemental screening with contrast-enhanced MRI in addition to standard-of-care annual screening mammography. Yala et al and others (see Supplementary Material 1), have developed DL models using mammograms^10,44,45^ (or MR images^12^) that outperform the Tyrer–Cuzick model (Figure 3), and that have been externally validated on large and diverse data sets from the United States, Europe and Asia.^10^ A prospective trial (*ScreenTrustMRI,* NCT04832594) is currently recruiting, which will evaluate use of one commercial AI tool and one in-house academic tool, to predict future breast cancer risk based on mammography images, and thereby optimize the triage of women to supplemental screening with breast MRI.

Going one step further, Manley et al^46^ demonstrated that their DL breast cancer risk score tool is modifiable, and that chemoprevention can decrease risk. DL tools have also been developed to automate the assessment of mammographic breast density, and have been clinically implemented at both academic and clinical radiology centers.^47,48^

## Ultrasound

Breast ultrasound can be used as both a supplemental screening modality (where it increases the cancer detection rate over mammography alone, particularly in women with dense breasts), and as a diagnostic tool in the work-up of mammographic or clinical findings.^49^ Unfortunately, in many cases, ultrasound demonstrates low specificity and prompts unnecessary biopsies. It also has high interreader variability.^50^ In an effort to boost the diagnostic accuracy of ultrasound, DL methods have been developed for breast ultrasound lesion segmentation, lesion detection, and lesion classification, for both automated and handheld ultrasound. Automated breast ultrasound generates thousands of images per patient exam, and so DL tools are particularly needed for lesion detection and to reduce interpretation time.^51^ DL-based segmentation methods are state of the art, outperforming conventional computerized methods.^51–53^ DL has also been applied to lesion detection and classification,^54–62^ with several reader studies reporting DL models that are equivalent or superior to radiologists,^49,62,63^ although in most of these studies, DL models were compared against general radiologists without subspecialty training in breast imaging, small data set sizes were used, and data were from a single institution.^58,62^ As such, more work is needed to demonstrate the generalizability of these models.

**Figure 4.**
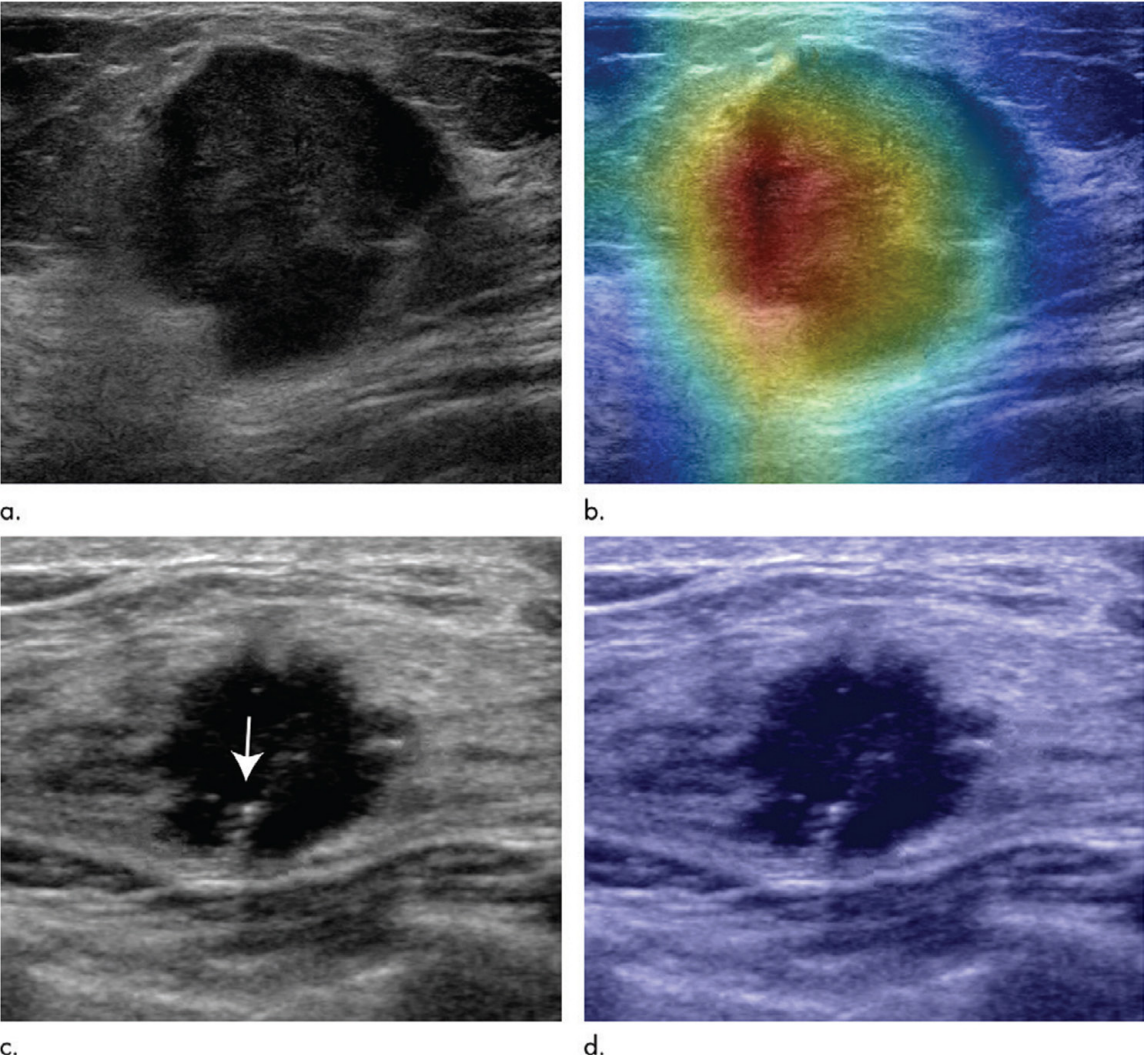
2D visualization of primary breast cancer in ultrasound images, and DL-aided prediction of clinically positive and negative lymph node metastasis.^58^ Here, DL enabled accurate prediction of positive lymph node metastasis in 67-year-old females (**a, b**) and negative lymph node metastasis in 46-year-old females (**c, d**). Reprinted by permission from Radiology,^58^ copyright Radiological Society of North America 2020. DL, deep learning.

DL for breast ultrasound has also been explored as a prognostication tool. Zhou et al^58^ and Zheng et al^64^ used ultrasound images of primary breast tumors to predict the presence of axillary node metastases, with AUCs up to 0.90. Figure 4 illustrates the use of DL to predict axillary nodal metastases using ultrasound images of primary breast cancer.

## Magnetic resonance imaging

Breast MRI is the most sensitive tool currently available for breast cancer detection,^65^ and is an indispensible tool in the screening of high risk women, in evaluating extent of disease, in assessing treatment response, and as a problem solving tool in challenging diagnostic situations. However, its use is often limited by high price tag and long exam time. High resolution dynamic contrast-enhanced MRI is an information-rich imaging modality with different imaging sequences (*e.g. T*_1_W, *T*_2_W, DWI, dynamic pre- and post-contrast imaging) reflecting different aspects of the underlying pathophysiology (*e.g.* water content, vascular permeability, etc.). This data-richness gives DL real potential not just to automate simple breast MR interpretation tasks, but to learn new patterns that uncover new connections between imaging and disease, opening new avenues for personalized medicine. To date, DL has been applied to breast MR image segmentation, lesion detection, risk prediction, and treatment response.^66^

### Segmentation, lesion detection and lesion classification

Today, DL is considered the state-of-the-art method for 3D segmentation of breast MRI images, including segmentation of the whole breast,^67,68^ fibroglandular tissue (FGT),^69,70^ and mass lesions^71,72^ (*see* Supplementary Material 1).

**Figure 5.**
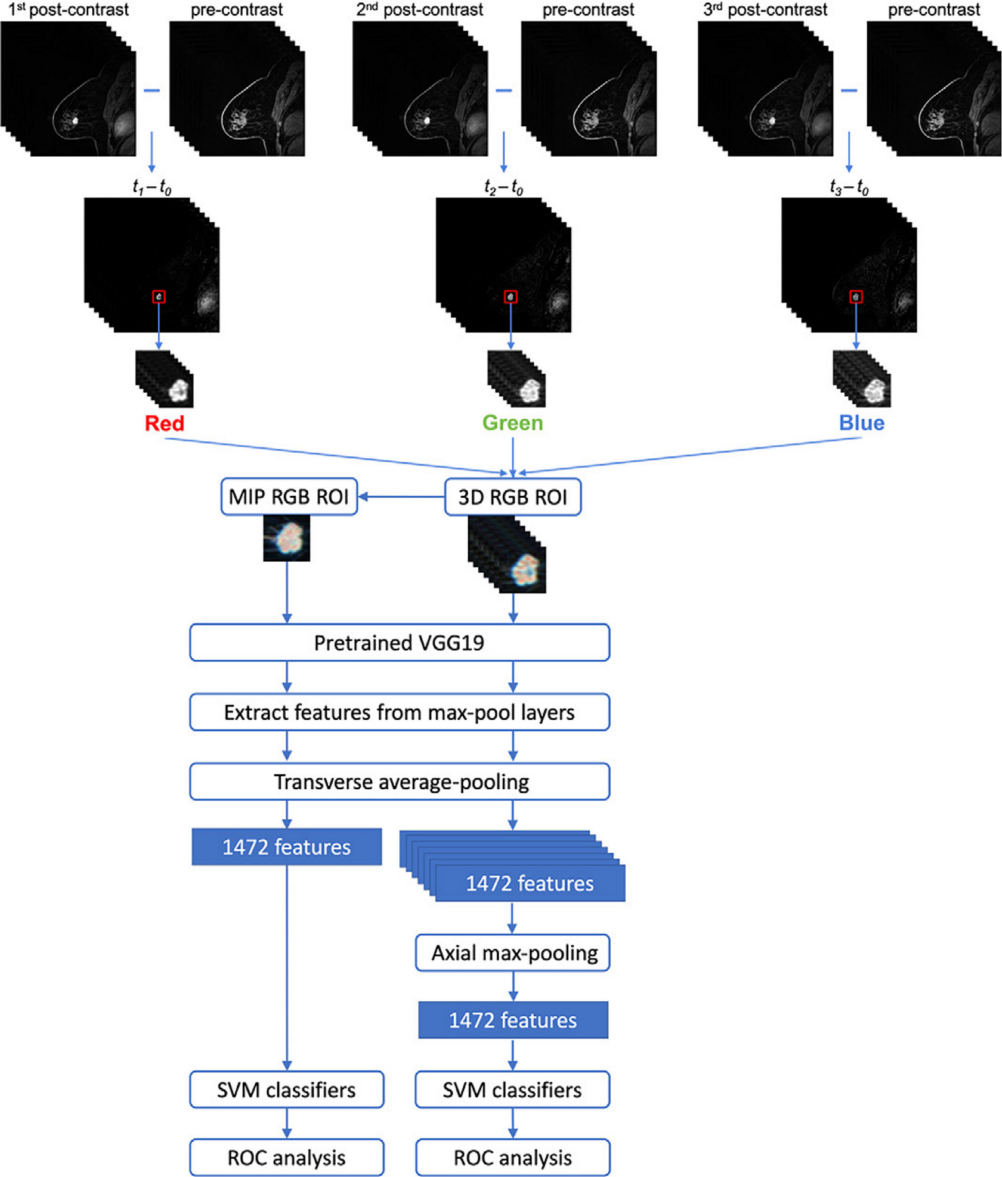
An illustration of combined DL feature extraction methods with traditional machine learning classifiers for lesion classification from cropped ROI’s of MIP and 3D RGB of subtraction images of DCE MRI sequences.^74^ The top layer illustrates the construction of the cropped ROI’s from the DCE MRI sequences. MIP and 3D RGB features were integrated with max-pooling and then passed to a machine learning classifier. Reprinted by permission from Radiology: Artificial Intelligence,^74^ copyright Radiological Society of North America 2021. DCE, dynamic contrast-enhanced; DL, deep learning; MIP, maximum intensity projection; ROI, region of interest.

Several groups have harnessed DL for both the detection and classification of lesions on breast MRI^73–81^ (see Supplementary Material 1 for details). The large size of a 4D breast MRI dataset makes *en mass* model training computationally difficult. Hence, breast MRI DL models hinge on imaging pre-processing pipelines that distill clinically relevant spatial and temporal information. The most popular (and easiest) approach is to collapse a 4D data set into a 2D maximum intensity projection (MIP) of the subtraction image (post-contrast minus pre-contrast), enabling the use of standard 2D CNN architectures for model training.^73–76,78,82^ Other groups have experimented with different approaches, including: (i) using a “MIP” of CNN features,^73,74^ (ii) using 3D lesion ROIs, not whole images, thus reducing data size requirements and enabling use of a 3D CNN,^76^ (iii) incorporating multiparametric information (*e.g.* T2, DWI),^76–78^ and (iv) fusing DL feature extraction methods with traditional machine learning classifiers.^74,76,77^ Figure 5 depicts a fused architecture integrating DL feature extraction and machine learning classifiers for lesion classification from subtraction images of dynamic contrast-enhanced (DCE) MRI sequences.^74^ Several papers show that DL methods for breast MRI outperform traditional machine learning methods, particularly as training data set sizes increase.^25^ Of the many studies published on DL for breast MRI, only a few include reader studies (two showing that DL models performed similarly to humans,^75,76^ and one showing that the DL model was inferior^71^). Of note, readers in these studies are not always radiologists, let alone fellowship-trained breast radiologists, and data set sizes are small. Additional larger and multicenter reader studies are needed to determine how DL models for breast MRI compares with human experts. In fact, small data set size is a limitation of all breast MRI DL papers to date, with the largest studies including only a couple thousand breast MRI exams. Breast MRI exams are also notoriously hard to curate, given the myriad sequences and the variations in protocol parameters and naming conventions even within the same institution.

### Risk prediction and treatment response

Background parenchymal enhancement (BPE) is a qualitative measure of normal breast tissue enhancement after intravenous contrast administration. Similar to breast density, BPE is included in a radiologist’s breast MRI report both to give information about whether the sensitivity for cancer detection is limited by BPE, and because BPE is a risk factor for breast cancer.^83^ Radiologists demonstrate significant interreader variability in categorizing BPE. DL has been applied both to segment^84^ and to classify BPE on breast MRI,^85^ enabling full automation and standardization of this process.

Additionally, in work analogous to that done with mammography, DL has also been used to predict 5-year breast cancer risk from the breast MRI MIP image directly,^12^ outperforming the standard-of-care Tyrer–Cuzick model.

The richness of breast MRI data makes it particularly well-suited for more complex DL-based prognostication. To create a virtual biopsy tool, DL with breast MRI images has been developed to predict breast tumor molecular subtypes using pathology ground truth of luminal A, luminal B, HER2+, and basal subtypes.^86–88^ Other groups have used DL to predict Oncotype Dx Recurrence Score using breast MRI data.^89^ DL has also been applied to the prediction of axillary nodal status using breast MR images of the primary tumor, with high cross-validation accuracy.^90–92^

Finally, DL techniques to predict patient response to neoadjuvant chemotherapy is an area of high interest. The landmark I-SPY 2 trial found that combinations of MRI features can predict pathologic treatment response using basic logistic regression analysis with AUCs of 0.81.^93^ Several groups are now working to improve predictive power using newer machine learning techniques, and with a combination of pre- and post- neoadjuvant chemotherapy MRI images.^13,14,91,94^ Much of this work is still preliminary, with small data sets from single institutions, although over the coming years, larger efforts in this area could personalize and optimize cancer therapy.

## Challenges and future directions

Since 2016, there has been exponential growth in the application of DL methods to all aspects of breast imaging. Still, more work is needed in several key areas.

First, large multi-institutional *prospective* trials, conducted by independent third parties, are needed to assess whether AI tools will work as expected in clinic. Retrospective and small reader studies show that AI mammography tools perform at or beyond the level of expert radiologists, and several AI decision-support products have already gained FDA approval.^95^ But prior to responsible clinical use, rigorous evaluation of these tools in a prospective setting is imperative.

AI mammography tools have been retrospectively validated in a few large multi-institutional external validation studies, but similar validation work is needed for ultrasound and especially for MRI. AI model development for breast MRI has been encouraging, but it is important to note that almost all published studies used small data sets and were from a single institution without external validation. DL breast MRI projects can be especially challenging since the breast MRI protocols are so variable across institutions, and even within the same institution over time. Still, there is a wealth of information within a breast MRI exam, and it remains a promising area of inquiry with potential to identify new and better ways to personalize the management of breast cancer patients to maximize therapeutic benefit and minimize harm.

In the realm of mammography, more technical work is needed to optimize AI tools for DBT. Radiologists detect more cancers and have fewer callbacks when using DBT compared to 2D mammography, and so it follows that AI-enhanced DBT should outperform AI-enhanced 2D mammography. But this is not yet the case. Developing state-of-the-art AI for DBT is a technically challenging pursuit. DBT exam sizes are much larger than 2D mammography, which translates into markedly increased computational costs during training which can pose technical limitations. Additionally, DBT image post-processing is even less standardized across vendors than 2D digital mammography, with significant variations in both acquisition technique (*i.e.* hardware) and in reconstruction technique (*i.e.* software). Available DBT data sets also tend to be smaller. Still, a number of recent studies have reported encouraging results, and also have an eye towards decreasing interpretation times.

As more AI tools are developed with potential for clinical translation, it is essential to tackle the associated ethical, medicolegal and regulatory issues. This is particularly important for standalone AI tools that independently interpret breast imaging exams (*i.e.* where no human radiologist looks at the images).^81^ On the ethical front, there are many unanswered questions. In which circumstances are clinicians obligated to inform patients about the use of AI tools in their clinical work-up? It might be particularly important in situations where AI acts as a “black box,” where clinicians act on an AI tool output but do not understand how the algorithm arrived at the conclusion. Who is liable when the AI tool misses a cancer? How much human oversight should be required? Algorithmic biases are also an ethical concern. AI models perform better on images that resemble images in the training data set, and so ongoing vigilance is needed to handle potentially underrepresented subgroups in the training data (*e.g.* racial groups, vendors, etc.). More work is also needed to improve the robustness of image normalization techniques, so that DL models can better generalize to data across institutions with different imaging hardware or imaging post-processing software. Finally, it is essential to develop new regulatory frameworks for rigorous AI quality assessment. This might include a regular schedule of AI algorithm quality control testing (similar to how imaging hardware undergoes regular quality control testing), as well as occasional fine-tuning of the algorithm prevent model performance from deteriorating over time.

Finally, one of the most striking aspects of this literature to date is the lack of improved algorithm performance when images at multiple prior timepoints are used.^30^ It is well known that the diagnostic accuracy of breast imagers markedly improves with the availability of prior mammograms, and yet the algorithms developed to date are not able to show similar improvements. This is clearly an area ripe for technical development.

## Conclusion

DL tools for breast imaging interpretation are being developed at a rapid pace and are likely to transform the clinical landscape of breast imaging over the coming years. Notably, DL mammography tools for breast cancer detection and breast cancer risk assessment demonstrate performance at or above human-level, and prospective trials are warranted to pave the way for clinical translation. Other work on DL for breast imaging opens up new possibilities for disease prognostication and personalized therapies. As DL tools are incorporated into clinical practice, however, regulatory oversight is needed to avoid algorithmic biases, prevent AI “performance drift”, and to address the unique ethical, medicolegal, and quality control issues that DL algorithms present.
